# Validity studies of the scale of positive and negative perceptions about alcohol effects

**DOI:** 10.11606/s1518-8787.2020054001811

**Published:** 2020-05-22

**Authors:** Leandro S. Almeida, Joana R. Casanova, María Fernanda Páramo Fernández, Caroline Tozzi Reppold, Maria Soledad Rodriguez Gonzalez

**Affiliations:** I Universidade do Minho Instituto de Educação Centro de Investigação em Educação Braga Portugal Universidade do Minho. Instituto de Educação. Centro de Investigação em Educação. Braga, Portugal; II Universidade de Santiago de Compostela Faculdade de Psicologia Santiago de Compostela Espanha Universidade de Santiago de Compostela. Faculdade de Psicologia. Santiago de Compostela, Espanha; III Universidade Federal de Ciências da Saúde de Porto Alegre Porto AlegreRS Brasil Universidade Federal de Ciências da Saúde de Porto Alegre. Porto Alegre, RS, Brasil

**Keywords:** Young Adult, Higher Education, Alcohol drinking, Health Risk Behaviors, Surveys and questionnaires, Validation Studies

## Abstract

**OBJECTIVE:**

To describe the process of elaboration and validation of the Scale of Perceptions about Alcohol Consumption in Higher Education Students in a Portuguese sample, considering the relationship between alcohol use rates and students’ perceptions about the effects of this consumption.

**METHODS:**

The validation study included 531 Portuguese college freshmen who answered the instrument, which is composed of five items that express positive perceptions and five items that express negative perceptions about the effects of alcohol consumption.

**RESULTS:**

Evidence of content validity, internal structure and external variables were obtained. The results of the factor analysis confirm the distribution of positive and negative perceptions by two different factors according to the theoretical model. Adequate internal consistency indexes were obtained for each dimension. The data obtained showed expected correlations between the perceptions and consumption behaviors of the students, indicating evidence of criterion validity of the scale. Moreover, the study showed that different consumption patterns between men and women, with higher alcohol consumption in the students’ households and restaurants or cafés by male students, in addition to the similarity in the consumption pattern between the two genders in parties and bars or nightclubs.

**CONCLUSION:**

The data obtained show the validity of the instrument. In the discussion, the article presents considerations about the responsibility of higher education institutions in the prevention and reduction in consumption rates among their students.

## INTRODUCTION

Alcohol consumption is considered a serious social and public health problem by the World Health Organization (WHO), with a particularly high rate in the most economically developed countries in Europe and the Americas^1^, especially among the academic population. In Brazil, official data of the 1st National Survey on Alcohol Use among university students showed that 67% of men and 56% of women reported having consumed alcohol in the last 30 days, suggesting a pattern of recurrent use. Young people between 18 and 24 years old reported a higher rate of consumption when compared with other age groups, whether in life, in the last 12 months or in the last 30 days. Among respondents under 18 years of age, 79.2% said having already ingested alcohol and 46.4% reported at least one occasion of high consumption (binge drinking) in the last 12 months^2^. Nevertheless, the survey also showed that only 27% of Brazilian higher education institutions developed some modality of prevention program or project and/or guidance and/or assistance to students regarding alcohol use^2^.

In Portugal, excessive alcohol consumption is also a public health problem among university students, particularly among those of the first year. WHO^3^ indicates that Portugal is one of the countries with the highest annual alcohol consumption among the adult population aged 15 years or over (12.9 L of pure alcohol/person), higher than 10.9 L in Europe and twice the world average consumption. According to the latest National Health Survey^4^, 67.3% of young Portuguese between 15 and 24 years old reported having a risk consumption pattern of alcohol consumption in the prior 12 months.

Studies point out the early age of consumption as a strong predictor of continuity in young adults and adults^5,6^ and indicate that the earlier this consumption, the greater its impact on psychopathological symptomatology^7,8^. They also show that the high consumption pattern among university students may be related to difficulties of students in adapting to higher education, especially among those who start living away from the family, which requires an autonomous management of their daily activities, academic responsibilities and even their economic resources. When experiencing these difficulties, some students initiate or increase alcohol consumption expecting it will facilitate the establishment of new interpersonal relationships and friendships or the overcoming of loneliness, anxiety and possible depressive symptoms^6,9^.

However, epidemiological data on alcohol consumption in university students show the organic, cognitive and emotional harmful effects of high alcohol consumption^1,8,9^. In addition to disturbances in academic activities such as absences from classes, non-delivery of activities within the established time frame or reduction of academic performance^10^, high consumption is associated with risk behaviors for the individual and his peers, particularly when expressed in antisocial, violent and inappropriate behaviors^13,14^.

Alcohol consumption in higher education students is associated with their own perceptions of its effects. Negative perceptions may assume a protective (dodge) function^15^and positive perceptions favor the development of attitudes and practices of greater tolerance to its effects^16,17^. Some studies also show that positive attitudes and expectations regarding alcohol consumption are related to earlier ages of initiation and continued consumption^18^, as well as a minimized perception of its negative effects^19^. Expectations that alcohol consumption is related to pleasure and university social life itself are also frequent^6^. Then a “culture of consumption” is established in student meetings and academic festivities^20^. Thus, in the adaptation phase to the university, alcohol intake can emerge as a resource for social and academic integration. High levels of stress and low self-esteem due to difficulties in academic adaptation may lead some students to become abusive alcohol users, presenting poorly reflective decision-making processes about their consumption^21^.

In the university context, correcting erroneous perceptions about alcohol use among students can be an effective strategy of health promotion^24,25^. If positive expectations of alcohol can induce excessive consumption, preventive programs can reduce such expectations in students^25,26^. By assuming that abusive consumption is associated with social stigmas and socially disruptive behaviors and values that often make it difficult to adhere to clinical treatments^27^, the evaluation of positive and negative perceptions about it can early point university students with a greater propensity for alcohol abuse.

We think it is necessary to offer reliable and valid instruments for the evaluation of positive and negative perceptions regarding alcohol consumption by students to support research and data for a consequent intervention. Previous difficulties in delimiting a set of items clearly differentiating these perceptions, particularly regarding attitudes and behaviors related to the negative ones, led us to construct a new scale using the statements of the students in the elaboration of the items^28^. Thus, this article describes the validation of a scale of positive and negative perceptions of alcohol consumption by testing the dimensionality and consistency of its items and appreciating its relationship with effective consumption behaviors by students. Considering the particular difficulties in its transition and academic adaptation, our study exclusively includes first-year university students.

## METHODS

### Participants

The sample consisted of 531 students, between 17 and 21 years old (mean = 18.50; standard deviation [SD] = 0.86). Regarding gender, 55% were women. Students of other years or over 21 years old were excluded. They attended courses in different areas: engineering (21.0%), sciences (24.1%), humanities (9.9%), nursing (16.3%), medicine (5.4%), arts (5.6%) and education (17.6%). Considering the policy of *numerus clausus* in Portugal (by which students are admitted to institutions and courses according to their application grade, considering the average of evaluations in secondary education and university access exams), 70.6% of students reported attending the course of their first choice. This sample was collected from a public institution of higher education in the northern region of Portugal. Seeking to diversify the areas of the courses attended, this is a convenience sample taken from the universe of 3,200 students who entered this university.

A second sample was withdrawn from the broader one, which also answered a questionnaire on alcohol consumption habits. This subsample was obtained based on the students’ consent to answer a set of items about their consumption patterns which contained information that was more personal and could be some discomfortable. For this analysis, the sample consisted of 237 students, with a mean age of 18.62 years (SD = 0.86). This sample continued to be composed mostly of female students, however, in an even higher percentage (79%).

### Instrument

The *Escala de Percepções sobre o Consumo de* Álcool *em Estudantes do Ensino Superior* (EPCAEES – Scale of Perceptions about Alcohol Consumption in Higher Education Students) consists of 10 situations or items in Likert type response format, ranging from 1 (totally disagree) to 6 (totally agree). Table 1 showed these items divided equally into two dimensions, according to the positive or negative tone of the perceived effects.

**Table 1:** Descriptive statistics of the score of items and factor weights in both factors.

Items	Mean	SD	Factor loadings	

			Negative perceptions	Positive perceptions
Leads to aggressive and unsociable behaviors (8)	3.49	1.29	0.84	
Creates dependency and leads to more consumption (9)	3.49	1.40	0.75	
It disrupts public order or create problems with the police (4)	3.14	1.27	0.72	
Causes memory lapses/loses awareness (5)	2.75	1.20	0.62	
Negatively affects learning and academic performance (1)	2.93	1.43	0.54	
Increases fun (3)	3.70	1.47		0.85
Allows me to spend better time (10)	3.11	1.34		0.84
Makes it easier to have meaningful life experiences (7)	3.06	1.44		0.79
Helps relieving stress or tensions (6)	3.67	1.36		0.71
Uninhibits me and facilitates contact with other students colleagues (2)	3.74	1.48		0.69

SD: standard deviation

Note: The number in front of each item indicates its position in the final version of the scale.

To elaborate the items, two groups were formed with students from science and humanities courses invited to express, in their own words, the reasons that lead them to consume or avoid alcohol. Thus, among the positive perceptions associated with consumption, the items refer to personal disinhibition, social interactions or stress relief. Regarding perceptions about the negative effects of alcohol consumption, the items are related to learning impairments, disruptive behavior or dependence.

A second part of the evaluation protocol questioned students about their alcohol consumption habits, including age of onset of consumption, frequency of consumption in general, frequency of consumption at home or household, frequency of consumption in cafés or restaurants and frequency of consumption at parties, bars or clubs. In all items related to attendance, students answered through a scale from 1 to 5, corresponding to “never,” “once or less per month,” “two to four times a month,” “two to three times a week” and “four or more times a week”). Students were also asked about how often they take six or more doses of alcohol on a single occasion (binge drinking) (0 = never; 1 = once or twice a year; 2 = once or twice a semester; 3 = once or twice a month; 4 = once or twice a week) and if entering higher education increased alcohol consumption (1 = yes; 2 = no).

### Data Collection Procedures

The scale was applied in the classroom by two psychologists from the institution informed of the nature and objectives of the study, in school times assigned by the teachers. The students were informed of the objectives of the study. All participants signed an informed consent form and had their confidentiality rights ensured, following the ethical standards of the Helsinki Declaration for human investigation. Only students that agreed to complete the questionnaire about consumption patterns provided their student number, thus enabling to pair the scale of perceptions with the form of consumption habits. All students present in the classes agreed to answer the scale of the perceptions; however, only one subsample of students consented to give elements about their consumption habits. The filling time ranged from five (sample 1) to ten minutes (sample 2), according to the number of instruments.

### Data Analysis Procedures

In a first analysis of the data, the missing values were replaced by the mean score of each factor, and the scores in the items formulated by the negative were reversed. The dimensionality of the scale was analyzed using confirmatory factor analysis (CFA), with the LISREL 9.3 program, and the parameters were estimated by maximum likelihood from the matrix of correlations between items. The evaluation of the model adjustment considered several indices: the chi-square value (χ^2^) divided by the degrees of freedom (CMIN), the mean quadratic error of approximation (RMSEA), the comparative fit index (CFI) and the residual quadratic mean root (RMR). According to Hu and Bentler^29^, RMSEA < 0.06, CFI > 0.95 and RMR < 0.08 are indicators of an acceptable fit. The reliability of the scale scores was estimated by Cronbach’s α coefficient. To analyze the external evidence of validity, the correlations between the scores of the perception scale and the variables related to alcohol consumption were estimated, controlling participants’ gender.

## RESULTS

Analyzing the structural validity of the scale items, we found that the perceptions related to alcohol consumption are split into two dimensions, that is, the five negative items saturate in one factor and the five positive items saturate in another. The two-dimensional oblique structure presents adequate adjustment indices in CFA (CMIN = 3.23; RMSEA = 0.06; CFI = 0.97; RMR = 0.05) and all estimated parameters were significant (p < 0.001). Table 1 shows the mean, standard deviation and saturations or factorial weights in the respective factor (negative perceptions and positive perceptions) for each item.

The results obtained allow us to verify that, in general, the items associated with positive perceptions of alcohol consumption obtained higher averages, and these values were particularly high in the item “Uninhibits me and facilitates contact with other college students.” The mean in the item “Causes memory lapses/loses awareness” had the lowest index of agreement. On the other hand, all items present saturations higher than 0.50 in the respective factor, being higher for items of positive perceptions.

Table 2 shows the distribution of the results in the two dimensions of the scale (minimum and maximum, mean and standard deviation), as well as Cronbach’s α coefficients and the correlations obtained between the scale dimensions.

**Table 2:** Distribution of results in the two dimensions of the scale, Cronbach’s α coefficient and correlations between the dimensions.

	Min.	Max.	Mean	SD	Cronbach’s α	Perceptions	

						Negative	Positive
Negative perceptions	5	30	15.79	5.02	0.82		
Positive perceptions	5	30	17.28	5.85	0.88	0.41^a^	

SD: standard deviation

^a^ p < 0.001

The mean scores in the positive perceptions were significantly higher than in the negative ones when the mean differences were estimated using the *t*-test for paired samples (t_530_ = 5.66; p < 0.001). The correlation between the two factors was moderate and significant (r = 0.412, p < 0.001). The internal consistency of the items for the two dimensions was high, being higher in the dimension of positive perceptions (α = 0.88).

Table 3 shows the consumption habits in the total subsample and in the subsamples subdivided according to the gender of the students based on the data referring to the second sample to study the criterion validity of the scale.

**Table 3:** Alcohol consumption in the total sample and differentiated by gender.

	Total (n = 237)		Male (n = 49)		Female (n = 188)	
		
	Mean	SD	Mean	SD	Mean	SD
AgeBeggining	15.35	1.91	14.96	2.57	15.46	1.70
FreqConsAlcohol	2.61	0.84	2.88	1.50	2.54	0.76
FreqConsHouse	1.66	0.86	2.00	1.08	1.57	0.77
FreqConsCafés	2.02	0.95	2.31	0.98	1.96	0.92
FreqConsParties	2.49	0.86	2.63	0.99	2.46	0.82
BingeDrinking	1.29	1.25	1.37	1.33	1.27	1.23
IncreasedConsumption	1.53	0.50	1.63	0.49	1.51	0.50
PercPositives	18.09	5.79	18.12	5.15	18.09	5.96
PercNegatives	15.42	4.10	15.02	4.14	15.53	4.09

SD: standard deviation; AgeBeggining: age of first consumption; FreqConsAlcohol: frequency of consumption of alcohol; FreqConsHouse: frequency of alcohol consumption at home; FreqConsCafés: frequency of alcohol consumption in cafés, FreqConsParties: frequency of alcohol consumption at parties; BingeDrinking: consumption of six or more doses of drink on a single occasion; IncreasedConsumption: increased alcohol consumption after entering higher education; PercPositives: positive perceptions of alcohol consumption; PercNegatives: negative perceptions of alcohol consumption.

The results point to a higher alcohol consumption by male students when analyzing the mean differences through the *t*-test for independent samples (t_235_ = 2.52; p = 0.01), either at home (t_235_ = 3.14; p = 0.002) or in cafés and restaurants (t_235_ = 2.32; p = 0.02). This situation no longer occurs in the item “consumption at parties” (t_235_ =1.27; p = 0.21).

Table 4 presents the correlations between alcohol consumption variables and students’ positive and negative perceptions of alcohol effects. Partial correlations controlling participants’ gender were also estimated due to the unequal distribution between the genders and the relationship of this variable with the frequency of consumption.

**Table 4:** Correlations between perceptions of alcohol effects and variables of consumption, without and with control of the variable gender.

Frequency of alcohol consumption	Perceptions	

	Negative	Positive
No control of the variable gender		
AgeBeggining	-0.139^a^	-0.207^b^
FreqConsAlcohol	0.332^c^	0.437^c^
FreqConsHouse	0.069	0.163^a^
FreqConsCafés	0.263^c^	0.433^c^
FreqConsParties	0.338^c^	0.508^c^
BingeDrinking	0.292^c^	0.305^c^
IncreasedConsumption	-0.316^c^	-0.528^c^
With control of the variable gender		
AgeBeggining	-0.146^a^	-0.208^b^
FreqConsAlcohol	0.345^c^	0.442^c^
FreqConsHouse	0.081	0.166^a^
FreqConsCafés	0.274^c^	0.438^c^
FreqConsParties	0.344^c^	0.509^c^
BingeDrinking	0.295^c^	0.305^c^
IncreasedConsumption	-0.313^c^	-0.531^c^

SD: standard deviation; AgeBeggining: age of first consumption; FreqConsAlcohol: frequency of consumption of alcohol; FreqConsHouse: frequency of alcohol consumption at home; FreqConsCafés: frequency of alcohol consumption in cafes, FreqConsParties: frequency of alcohol consumption at parties; BingeDrinking: consumption of six or more doses of drink on a single occasion; IncreasedConsumption: increased alcohol consumption after entering higher education; PercPositives: positive perceptions of alcohol consumption; PercNegatives: negative perceptions of alcohol consumption.

^a^ p < 0.05

^b^ p < 0.01

^c^ p < 0.001

The results suggest that all consumption variables were significantly statistically related to the perceptions of alcohol effects, except when home consumption and negative perceptions are crossed. It also occurs when the gender variable is assessed.

**Figure 1:**
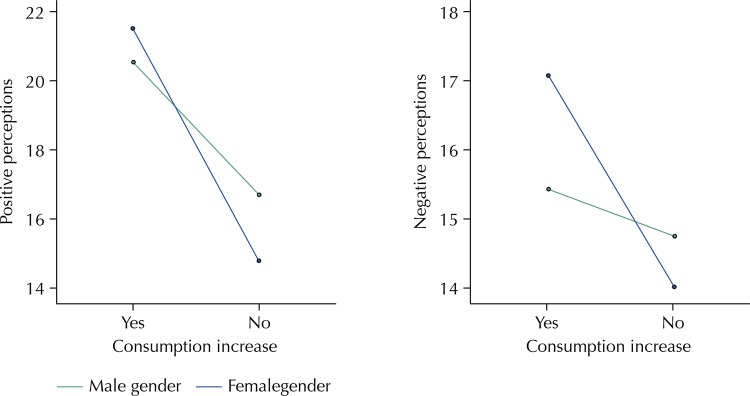
Relationships between perceptions of the effects of alcohol consumption and consumption after entering university according to gender.

Finally, since the variable “increase in consumption” (“Did entering higher education increase your alcohol consumption?”) is nominal, the differences between the perceptions were analyzed based on students who answered “yes” or “no.” Figure 1 shows that the perceptions, both positive and negative, differed between these two groups of students in the F test of variance analysis (F_1.233_ = 42.73; p < 0.001 and F_1.233_ = 8.35; p < 0.01, respectively). The effect of increased consumption, estimated using the Eta Square value, was higher in positive perceptions (η^2^ = 0.155) than in negative ones (η^2^ = 0.035). On the other hand, there was no significant effect of the variable “gender” or the interaction between the two variables.

## DISCUSSION

Excessive alcohol consumption is a public health problem, since they represent significant costs of public health systems. Abusive or high consumption is associated with mortality and different cognitive, affective and behavior dysfunctions^1^. This problem deserves particular attention in adolescence and in the transition to adulthood, in which binge drinking is often seen^27^. An abusive pattern of alcohol consumption among higher education students may be associated with the demands in the transition and adaptation to academic life as sources of stress, emotional instability and psychosocial vulnerability^3,4,7,8^.

One of the important variables in the investigation and intervention in consumption behaviors is related to the perceptions of young people regarding the effects of alcohol. Higher consumption habits follow positive perceptions about it. Likewise, students tend to be more susceptible to the influence of peers and academic environments that stimulate alcohol intake if they underestimate the negative effects of alcohol consumption and have reduced rates of negative perceptions^30^. Thus, it is important to evaluate, since the earliest times in university, the perceptions of young people about such behavior, both positive and negative ones, given the specificity of both dimensions for an analysis of the impact on consumption habits. Alcohol consumption mostly occurs in group contexts and academic parties, in which positive perceptions become relevant, since they are socially constructed and possibly associated with greater effectiveness when the intervention is done in a group^27^.

Therefore, this article describes the process of elaboration of the Scale of Perceptions about Alcohol Consumption in Higher Education Students and the evidence of content validity, dimensionality and criterion addressing external variables with a sample of Portuguese students. Confirmatory factor analysis shows the existence of two dimensions, grouping the items of positive and negative perceptions, according to the theoretical framework. Likewise, high levels of internal consistency of the items were observed for each dimension, reaching values higher than 0.80. Moreover, the positive and negative perceptions evaluated are correlated with the behaviors and consumption habits of students, since the consumption is higher in parties and leisure spaces, both for male and female students, with a higher rate for male students when it occurs in the residence or cafés and restaurants. These data suggest a higher prevalence in male students in different contexts, with a higher propensity of consumption by female students in situations of social interaction, indicating consumption as an instrument that promotes social and academic integration^20,22^.

The results obtained emphasize the importance of comprehensive and articulated public policies, preferably preventive, focusing on education and health promotion or on increasing quality of life. The data show that the institutions of higher education should respond for the severity of the phenomenon of alcohol consumption. Their effort to alert to the physiological and psychosocial effects of such consumption and dependence, appealing to the autonomy and responsibility of young people, seems to be ineffective due to the students’ perceptions. The consultation services, including information and counselling actions, have failed to reduce the incidence of regular consumption and peaks in the festive moments. In a preventive logic and relating abusive consumption to weaknesses in the adaptation of students, it is important to reduce the frequency and intensity of situations of vulnerability, isolation and loneliness that some young people experience in higher education, as well as to ensure alternative contexts of social interaction not marked by substance consumption. Therefore, data from a study that uses a valid screening instrument for students’ perceptions about the effects of alcohol can contribute to the implementation of strategies for prevention and health promotion that are more effective and consistent with the reality of each university.
