# Supplementary material for: Validity studies of the scale of positive and negative perceptions about alcohol effects
Source: Rev Saude Publica. 2020 May 22;54:52. doi: 10.11606/s1518-8787.2020054001811 (PMC7244234; doi:10.11606/s1518-8787.2020054001811)
Supplement: Translation

# Translation: Estudos de validade da escala de percepções positivas e negativas sobre efeitos do álcool

**Authors:** Leandro S. Almeida, Joana R. Casanova, María Fernanda Páramo Fernández, Caroline Tozzi Reppold, Maria Soledad Rodriguez Gonzalez

**Affiliations:** I Portugal Universidade do Minho. Instituto de Educação. Centro de Investigação em Educação (CIEd). Braga, Portugal; II Espanha Universidade de Santiago de Compostela. Faculdade de Psicologia. Santiago de Compostela, Espanha; III Brasil Universidade Federal de Ciências da Saúde de Porto Alegre. Porto Alegre, RS, Brasil

**Keywords:** Adulto Jovem, Educação Superior, Consumo de Bebidas Alcoólicas, psicologia, Comportamentos de Risco à Saúde, Inquéritos e Questionários, Estudos de Validação

## Abstract

**OBJETIVO:**

Considerando a relação entre as taxas de uso de álcool e as percepções dos estudantes sobre os efeitos desse consumo, descrever o processo de elaboração e validação da Escala de Percepções sobre o Consumo de Álcool em Estudantes do Ensino Superior em uma amostra portuguesa.

**MÉTODOS:**

Participaram do estudo de validade 531 estudantes portugueses do primeiro ano do ensino superior que responderam ao instrumento, composto por cinco itens que expressam percepções positivas e cinco itens que expressam percepções negativas sobre os efeitos do consumo de álcool.

**RESULTADOS:**

Evidências de validade de conteúdo, de estrutura interna e baseadas em variáveis externas foram obtidas. Os resultados da análise fatorial confirmam a distribuição das percepções positivas e negativas por dois fatores diferenciados em acordo com o modelo teórico de partida. Índices adequados de consistência interna foram obtidos para cada dimensão. Os dados obtidos mostraram correlações esperadas entre as percepções e os comportamentos de consumo dos estudantes, indicando evidências de validade de critério da escala. Em acréscimo, o estudo indicou haver padrões de consumo diferenciados entre os sexos, registando-se maior consumo de álcool nas residências e nos restaurantes ou cafés por parte dos homens, além de similaridade entre os dois sexos no padrão de consumo em festas e bares ou discotecas.

**CONCLUSÃO:**

Os dados obtidos evidenciam a validade do instrumento. Na discussão dos dados, o artigo apresenta considerações sobre a responsabilidade das instituições de ensino superior na prevenção e redução das taxas de consumo entre a sua população estudantil.

## INTRODUÇÃO

O consumo de álcool é reconhecido pela Organização Mundial de Saúde (OMS) como um problema social e de saúde pública grave, com uma taxa particularmente elevada nos países economicamente mais desenvolvidos da Europa e das Américas^1^, especialmente entre a população acadêmica. No Brasil, dados oficiais do I Levantamento Nacional sobre Uso de Álcool entre universitários revelaram que 67% dos homens e 56% das mulheres afirmaram ter consumido álcool nos *últimos* 30 dias, o que sugere um padrão de uso recorrente. Jovens entre 18 e 24 anos relataram taxa maior de consumo em relação a outras faixas etárias, seja na vida, nos últimos 12 meses ou nos últimos 30 dias. Entre os entrevistados com menos de 18 anos de idade, 79,2% afirmaram já haver ingerido álcool e 46,4% relataram pelo menos uma ocasião de consumo elevado (*binge drinking*) nos últimos 12 meses^2^. Apesar disso, o levantamento mostrou também que apenas 27% das instituições de ensino superior brasileiras desenvolviam alguma modalidade de programa ou projeto de prevenção e/ou orientação e/ou assistência aos discentes em relação ao uso de álcool^2^.

Em Portugal, o consumo excessivo de álcool também é um problema de saúde pública entre universitários, em particular entre os estudantes do 1º ano. A OMS^3^ indica que Portugal é um dos países mundiais com maior consumo anual de álcool na população adulta de 15 ou mais anos (12,9 L de álcool puro/pessoa), superior aos 10,9 L na Europa e o dobro do consumo médio mundial, estimado em 6,2 L. De acordo com o último Inquérito Nacional de Saúde^4^, 67,3% dos jovens portugueses entre 15 e 24 anos afirmaram ter um padrão de consumo arriscado de consumo de bebidas alcoólicas nos últimos 12 meses.

Estudos apontam a idade precoce do consumo como um forte preditor da continuidade desse consumo em jovens adultos e adultos^5,6^ e indicam que quanto mais precoce esse consumo, maior o seu impacto em sintomatologia psicopatológica^7,8^. Evidenciam também que o padrão de consumo elevado entre universitários pode estar relacionado com dificuldades enfrentadas pelos estudantes na adaptação ao ensino superior, em especial entre aqueles que passam a viver afastados da família, tendo que gerir de forma autônoma suas atividades diárias, responsabilidades acadêmicas e, inclusive, seus recursos econômicos. Vivenciando essas dificuldades, alguns iniciam ou aumentam o consumo de álcool, na expectativa de que a bebida seja facilitadora do estabelecimento de novas relações interpessoais e amizades ou da superação da solidão, ansiedade e de eventuais sintomas depressivos^6,9^.

Contudo, dados epidemiológicos sobre consumo de álcool em universitários demonstram os efeitos nefastos de um consumo elevado em nível orgânico, cognitivo e emocional^1,8,9^. Para além de perturbações nas atividades acadêmicas, traduzidas nas faltas às aulas, não entrega de trabalhos no prazo estabelecido ou redução do rendimento acadêmico^10^, o consumo elevado é associado a comportamentos de risco para o próprio sujeito e para os seus pares, nomeadamente quando expresso em comportamentos antissociais, violentos e contrários à ordem pública^13,14^.

O consumo de álcool em estudantes do ensino superior aparece associado às suas percepções relativas aos efeitos de tal consumo, podendo as percepções negativas assumirem uma função protetora (de esquiva)^15^ e as percepções positivas favorecerem o desenvolvimento de atitudes e práticas de maior tolerância aos efeitos e consumo do álcool^16,17^. Alguns estudos mostram também que atitudes e expectativas positivas em relação ao consumo de álcool se relacionam a idades mais precoces de iniciação e a um consumo continuado^18^, bem como a uma percepção minimizada de seus efeitos negativos^19^, sendo frequentes as expectativas de que esteja relacionado ao prazer e à própria vida social universitária^6^. Nessa linha, encontra-se instituída uma “cultura do consumo” nas reuniões sociais estudantis e festividades acadêmicas^20^. Assim, na fase de adaptação à universidade, a ingestão de álcool pode surgir como recurso de integração social e acadêmica. Diante dos altos níveis de estresse e da baixa autoestima enfrentados em decorrência de dificuldades na adaptação acadêmica, alguns estudantes tornam-se consumidores abusivos de álcool, apresentando processos de decisão pouco reflexivos sobre seu consumo^21^.

Diante dessas tradições acadêmicas, corrigir percepções errôneas sobre o uso de álcool entre os estudantes pode ser uma estratégia de promoção da saúde no contexto universitário^24,25^. Se as expectativas positivas do álcool podem induzir um consumo excessivo, programas preventivos devem reduzir tais expectativas nos estudantes^25,26^. Assumindo que o consumo abusivo está associado a estigmas sociais e a condutas e valores socialmente disruptivos que muitas vezes dificultam a adesão a tratamentos clínicos^27^, a avaliação de percepções positivas e negativas sobre ele pode servir para uma sinalização precoce de universitários com maior propensão para o excesso.

Em apoio à investigação nessa área e de modo a subsidiar dados para uma intervenção consequente, faz-se necessária a oferta de instrumentos confiáveis e válidos para a avaliação das percepções positivas e negativas em relação ao consumo do álcool por parte dos estudantes. Dificuldades anteriores na delimitação de um conjunto de itens diferenciando de forma clara essas percepções, nomeadamente em termos de atitudes e comportamentos referentes às negativas, levou-nos à construção de uma nova escala, tomando as verbalizações dos próprios estudantes na elaboração dos itens^28^. Assim, este artigo descreve os procedimentos de validação de uma escala de percepções positivas e negativas de consumo de álcool testando a dimensionalidade e a consistência dos seus itens e apreciando a sua relação com comportamentos efetivos de consumo por parte dos estudantes. Tendo em vista as particulares dificuldades na sua transição e adaptação acadêmica, este estudo inclui exclusivamente estudantes universitários do 1º ano.

## MÉTODOS

### Participantes

A amostra, após a exclusão de alunos de outros anos da faculdade ou idade superior a 21 anos, foi formada por 531 estudantes do 1º ano, sendo 55% mulheres. A idade ficou compreendida entre 17 e 21 anos (média = 18,50; desvio-padrão [DP] = 0,86). Esses estudantes eram provenientes de cursos de diversas áreas científicas: engenharia (21,0%), ciências (24,1%), humanidades (9,9%), enfermagem (16,3%), medicina (5,4%), artes (5,6%) e educação (17,6%). No conjunto da amostra, levando em conta a política de *numerus clausus* em Portugal (pela qual os alunos são admitidos nas instituições e cursos em função da sua nota da candidatura, considerando a média das avaliações no ensino secundário e nos exames de acesso à universidade), 70,6% dos estudantes referiram frequentar o curso de sua primeira opção de escolha. Essa amostra foi recolhida numa instituição pública de ensino superior na região norte de Portugal. Trata-se de uma amostra de conveniência retirada do universo de 3.200 estudantes que ingressaram nessa universidade, procurando-se diversificar as áreas científicas dos cursos frequentados.

Dessa amostra mais ampla, retirou-se uma segunda amostra que respondeu também a um questionário sobre hábitos de consumo de álcool. Essa subamostra foi obtida com base na disponibilidade dos estudantes para responder a um conjunto de itens, contendo informações mais pessoais e susceptíveis a gerar algum desconforto, sobre os seus padrões de consumo. Para essa análise, a amostra foi formada por 237 estudantes, com média de idade de 18,62 anos (DP = 0,86). Essa amostra continuou sendo maioritariamente formada por estudantes do sexo feminino, mas desta vez em uma percentagem ainda mais elevada (79%).

### Instrumento

A Escala de Percepções sobre o Consumo de Álcool em Estudantes do Ensino Superior (EPCAEES) é formada por 10 situações ou itens em formato tipo Likert de resposta, cujas âncoras variavam de 1 (totalmente em desacordo) a 6 (totalmente de acordo). Esses itens, apresentados na Tabela 1, dividem-se equitativamente em duas dimensões, conforme a tonalidade positiva ou negativa dos efeitos percebidos.

**Tabela 1:** Estatísticas descritivas da pontuação dos itens da escala e pesos fatoriais nos dois fatores.

Itens	Média	DP	Pesos fatoriais	

			Percepções negativas	Percepções positivas
Leva a comportamentos agressivos e pouco sociáveis (8)	3,49	1,29	0,84	
Cria dependência e leva a consumir mais (9)	3,49	1,40	0,75	
Faz perturbar a ordem pública ou ter problemas com a polícia (4)	3,14	1,27	0,72	
Provoca lapsos de memória/perde-se a consciência das coisas (5)	2,75	1,20	0,62	
Afeta negativamente a aprendizagem e o rendimento acadêmico (1)	2,93	1,43	0,54	
Aumenta a diversão (3)	3,70	1,47		0,85
Permite passar melhor o tempo (10)	3,11	1,34		0,84
Facilita ter experiências de vida significativas (7)	3,06	1,44		0,79
Ajuda a aliviar o estresse ou as tensões (6)	3,67	1,36		0,71
Desinibe e facilita o contato com os colegas (2)	3,74	1,48		0,69

DP: desvio-padrão

Nota: O número à frente de cada item indica a sua posição na versão definitiva da escala.

Para elaboração dos itens, foram realizados dois grupos focais com estudantes provenientes de cursos de ciências e humanidades, convidando-os a expressar, por palavras do seu cotidiano, os motivos que os levam a consumir ou a evitar o consumo abusivo de álcool. Assim, entre as percepções positivas associadas ao consumo, os itens referem-se à desinibição pessoal, interações sociais ou alívio do estresse. Por sua vez, relativamente a percepções sobre os efeitos negativos do consumo de álcool, os itens estão relacionados a prejuízos na aprendizagem, problemas com a ordem pública ou criação de dependência.

Uma segunda parte do protocolo de avaliação utilizado questionava os estudantes sobre os seus hábitos de consumo de álcool, nomeadamente idade de início do consumo, frequência de consumo em geral, frequência de consumo em casa ou residências, frequência de consumo em cafés ou restaurantes e frequência de consumo em festas, bares ou baladas. Em todos os itens referentes à frequência, os estudantes respondiam por meio de uma escala de cinco âncoras (de 1 a 5, descritas como “nunca”, “uma vez ou menos por mês”, “duas a quatro vezes por mês”, “duas a três vezes por semana” e “quatro ou mais vezes por semana”). Em acréscimo, os estudantes foram questionados sobre a frequência com que tomam seis ou mais doses de bebida alcoólica em uma única ocasião (*binge drinking*) (0 = nunca; 1 = uma ou duas vezes por ano; 2 = uma ou duas vezes por semestre; 3 = uma ou duas vezes por mês; 4 = uma ou duas vezes por semana) e se o ingresso no ensino superior aumentou o consumo de bebidas alcoólicas (1 = sim; 2 = não).

### Procedimentos de Coleta dos Dados

A escala foi aplicada em sala de aula, em tempos letivos cedidos pelos professores, por dois psicólogos da instituição informados da natureza e objetivos do estudo. Os estudantes foram informados dos objetivos do estudo. Todos participantes deram o seu consentimento informado e tiveram seus direitos de confidencialidade assegurados, seguindo as normas éticas da Declaração de Helsinque para investigação com seres humanos. Apenas os estudantes que aceitaram preencher o questionário sobre os padrões de consumo forneceram seu número de estudante, de forma a permitir o emparelhamento da escala de percepções com a ficha dos hábitos de consumo. Todos os estudantes presentes nas aulas concordaram em responder à escala das percepções, contudo apenas uma subamostra se disponibilizou a dar elementos sobre os seus hábitos de consumo. O tempo de preenchimento variou entre cinco (amostra 1) e dez minutos (amostra 2), conforme o número de instrumentos a serem respondidos pelos estudantes.

### Procedimentos de Análise dos Dados

Em uma primeira exploração dos dados, os valores omissos (*missing*) foram substituídos pela pontuação média de cada fator, e as pontuações nos itens formulados pela negativa foram invertidas. A dimensionalidade da escala foi analisada por meio da análise fatorial confirmatória (AFC), com o programa LISREL 9.3, e a estimação dos parâmetros foi realizada pela máxima verossimilhança a partir da matriz de correlações entre itens. A avaliação do ajuste do modelo considerou diversos índices: o valor de qui-quadrado (χ^2^) dividido pelos graus de liberdade (CMIN), o erro quadrático médio de aproximação (RMSEA), o índice de ajuste comparativo (CFI) e a raiz média quadrática residual (RMR). Segundo Hu e Bentler^29^, valores de RMSEA < 0,06, CFI > 0,95 e RMR < 0,08 são considerados indicadores de um ajuste aceitável. A confiabilidade das pontuações da escala foi estimada pelo coeficiente α de Cronbach. Para analisar as evidências externas de validade, calcularam-se as correlações entre as pontuações da escala de percepções e as variáveis relacionadas com o consumo de álcool, controlando o sexo dos participantes.

## RESULTADOS

Centrando-nos na validade estrutural dos itens da escala, verificou-se que as percepções relativas ao consumo de álcool se repartem em duas dimensões, ou seja, os cinco itens negativos saturam num fator e os cinco itens positivos saturam noutro. A estrutura bidimensional oblíqua apresenta índices adequados de ajustamento na AFC ( CMIN = 3,23 RMSEA = 0,06; CFI = 0,97; RMR = 0,05) e todos os parâmetros estimados foram significativos (p < 0,001). Na Tabela 1, são apresentadas as estatísticas descritivas para cada item, nomeadamente a média, desvio-padrão e saturações ou pesos fatoriais no respectivo fator (percepções negativas e percepções positivas).

Os resultados obtidos permitem verificar que, em geral, os itens associados a percepções positivas do consumo de álcool obtiveram médias mais elevadas, sendo esses valores particularmente elevados no item “Desinibe e facilita o contato com os colegas”. A média no item “Provoca lapsos de memória/perde-se a consciência das coisas” apresentou o índice mais baixo de concordância. Por outro lado, todos os itens apresentam saturações superiores a 0,50 no respectivo fator, sendo esses coeficientes mais elevados para os itens de percepções positivas.

Na Tabela 2, é apresentada a distribuição dos resultados nas duas dimensões da escala (mínimo e máximo, média e desvio-padrão), assim como os coeficientes α de Cronbach e as correlações obtidas entre as dimensões da escala.

**Tabela 2:** Distribuição dos resultados nas duas dimensões da escala, coeficiente α de Cronbach e correlações entre as dimensões.

	Mín.	Máx.	Média	DP	α de Cronbach	Percepções	

						Negativas	Positivas
Percepções negativas	5	30	15,79	5,02	0,82		
Percepções positivas	5	30	17,28	5,85	0,88	0,41^a^	-

DP: desvio-padrão

^a^ p < 0,001

As pontuações médias nas percepções positivas foram significativamente maiores que nas negativas, quando calculadas as diferenças médias através do teste-t para amostras emparelhadas (t_530_ = 5,66; p < 0,001). A correlação entre os dois fatores foi moderada e significativa (r = 0,412, p < 0,001). A consistência interna dos itens para as duas dimensões foi elevada, sendo mais alta na dimensão das percepções positivas (α = 0,88).

Tomando os dados referentes à segunda amostra para estudo da validade de critério da escala, na Tabela 3 são apresentados os hábitos de consumo na subamostra total e nas subamostras subdivididas em função do sexo dos estudantes.

**Tabela 3:** Consumo de álcool na amostra total e diferenciada por sexo.

	Total (n = 237)		Masculino (n = 49)		Feminino (n = 188)	
		
	Média	DP	Média	DP	Média	DP
IdadeInício	15,35	1,91	14,96	2,57	15,46	1,70
FreqConsÁlcool	2,61	0,84	2,88	1,50	2,54	0,76
FreqConsCasa	1,66	0,86	2,00	1,08	1,57	0,77
FreqConsCafés	2,02	0,95	2,31	0,98	1,96	0,92
FreqConsFestas	2,49	0,86	2,63	0,99	2,46	0,82
BingeDrinking	1,29	1,25	1,37	1,33	1,27	1,23
AumentoConsumo	1,53	0,50	1,63	0,49	1,51	0,50
PercPositivas	18,09	5,79	18,12	5,15	18,09	5,96
PercNegativas	15,42	4,10	15,02	4,14	15,53	4,09

DP: desvio-padrão; IdadeInício: idade de primeiro consumo; FreqConsÁlcool: frequência de consumo de álcool; FreqConsCasa: frequência de consumo de álcool em casa; FreqConsCafés: frequência de consumo de álcool em cafés, FreqConsFestas: frequência de consumo de álcool em festas; BingeDrinking: consumo de seis ou mais doses de bebida em uma única ocasião; AumentoConsumo: aumento do consumo de álcool depois de ingressar no ensino superior; PercPositivas: percepções positivas do consumo de álcool; PercNegativas: percepções negativas do consumo de álcool.

Os resultados apontam para um maior consumo de álcool pelos estudantes do sexo masculino quando se analisam as diferenças médias através do teste-t para amostras independentes (t_235_ = 2,52; p = 0,01), seja em casa (t_235_ = 3,14; p = 0,002) ou nos cafés e restaurantes (t_235_ = 2,32; p = 0,02). Essa situação já não ocorre em relação ao consumo em festas (t_235_ =1,27; p = 0,21).

Na Tabela 4, são apresentadas as correlações entre as variáveis do consumo de álcool e as percepções positivas e negativas dos estudantes em relação aos efeitos do álcool. Como a amostra possui uma distribuição muito desigual entre os dois sexos e essa própria variável está relacionada com a frequência do consumo, foram calculadas também as correlações parciais controlando o sexo dos participantes.

**Tabela 4:** Correlações entre percepções dos efeitos do *álcool* e variáveis do consumo, sem e com controle da variável sexo.

Variáveis de consumo de álcool	Percepções	

	Negativas	Positivas
Sem controle da variável sexo		
IdadeInício	-0,139^a^	-0,207^b^
FreqConsÁlcool	0,332^c^	0,437^c^
FreqConsCasa	0,069	0,163^a^
FreqConsCafés	0,263^c^	0,433^c^
FreqConsFestas	0,338^c^	0,508^c^
BingeDrinking	0,292^c^	0,305^c^
AumentoConsumo	-0,316^c^	-0,528^c^
Com controle da variável sexo		
IdadeInício	-0,146^a^	-0,208^b^
FreqConsÁlcool	0,345^c^	0,442^c^
FreqConsCasa	0,081	0,166^a^
FreqConsCafés	0,274^c^	0,438^c^
FreqConsFestas	0,344^c^	0,509^c^
BingeDrinking	0,295^c^	0,305^c^
AumentoConsumo	-0,313^c^	-0,531^c^

IdadeInício: idade de primeiro consumo; FreqConsÁlcool: frequência de consumo de álcool; FreqConsCasa: frequência de consumo de álcool em casa; FreqConsCafés: frequência de consumo de álcool em cafés, FreqConsFestas: frequência de consumo de álcool em festas; BingeDrinking: consumo de seis ou mais doses de bebida em uma única ocasião; AumentoConsumo: aumento do consumo de álcool depois de ingressar no ensino superior; PercPositivas: percepções positivas do consumo de álcool; PercNegativas: percepções negativas do consumo de álcool.

^a^ p < 0,05

^b^ p < 0,01

^c^ p < 0,001

Os resultados sugerem que todas as variáveis de consumo se relacionaram de forma estatisticamente significativa com as percepções dos efeitos do álcool, excetuando quando se cruza o consumo em casa e as percepções negativas. Essa situação verifica-se também quando se controla a variável sexo.

Por último, como a variável aumento do consumo (“O ingresso no ensino superior aumentou o seu consumo de bebidas alcoólicas?”) é nominal, as diferenças entre as percepções foram analisadas com base nos estudantes que responderam sim ou não a esse aumento. Na Figura, observa-se que as percepções, tanto positivas como negativas, diferenciaram-se entre esses dois grupos de estudantes no teste F da análise de variância (F_1,233_ = 42,73; p < 0,001 e F_1,233_ = 8,35; p < 0,01, respectivamente). O efeito do aumento do consumo, estimado através do valor de Eta Square, foi maior nas percepções positivas (η^2^ = 0,155) que nas negativas (η^2^ = 0,035). Por outro lado, não foi verificado um efeito significativo da variável sexo, nem da interação entre as duas.

**Figura:** Relações entre percepções dos efeitos do consumo de álcool e consumo após ingresso acadêmico de acordo com o sexo.

## DISCUSSÃO

O consumo excessivo de álcool é um problema de saúde pública, do qual decorrem encargos relevantes nos sistemas públicos de saúde. O consumo abusivo ou elevado aparece associado à mortalidade e a disfunções diversas em nível cognitivo, afetivo e comportamental^1^. O problema merece particular atenção na adolescência e na transição do jovem para a idade adulta, na qual surgem frequentemente situações de *binge drinking*^27^. Um padrão abusivo de consumo de álcool entre estudantes do ensino superior pode estar associado com as exigências na transição e adaptação à vida acadêmica enquanto fontes de estresse, instabilidade emocional e vulnerabilidade psicossocial^3,4,7,8^.

Uma das variáveis importantes na investigação e na intervenção nos comportamentos de consumo relaciona-se com as percepções dos jovens relativas aos efeitos do álcool. Maiores hábitos de consumo acompanham percepções positivas sobre ele. Ao mesmo tempo, se os estudantes não valorizam os efeitos negativos do consumo de álcool e apresentam índices reduzidos de percepções negativas, tendencialmente estarão mais permeáveis à influência dos pares e dos ambientes acadêmicos que estimulam essa ingestão^30^. Neste sentido, ressalta-se a importância de se avaliar, desde os primeiros tempos na universidade, as percepções dos jovens sobre tal comportamento, tanto positivas quanto negativas, dada a especificidade de ambas as dimensões para uma análise do impacto nos hábitos de consumo. Como ele ocorre maioritariamente em contextos de grupo e festas acadêmicas, tais percepções ganham relevância, pois são construídas socialmente e podem estar associadas à maior eficácia quando a intervenção é feita em grupo^27^.

Nesse sentido, o presente artigo descreve o processo de elaboração da Escala de Percepções sobre o Consumo de Álcool em Estudantes do Ensino Superior e as evidências de validade de conteúdo, de dimensionalidade e de critério face a variáveis externas com uma amostra de estudantes portugueses. A análise fatorial confirmatória mostra a existência de duas dimensões, agrupando os itens das percepções positivas e das negativas, de acordo com o referencial teórico de partida. No mesmo sentido, foram observados níveis elevados de consistência interna dos itens para cada dimensão, atingindo valores superiores a 0,80. Acresce que as percepções positivas e negativas avaliadas se apresentam correlacionadas com os comportamentos e hábitos de consumo dos estudantes, que é maior em festas e espaços de divertimento, tanto para homens como para mulheres, registando-se maior por parte dos estudantes do sexo masculino quando ocorre na residência ou cafés e restaurantes. Esses dados sugerem maior prevalência nos estudantes do sexo masculino nos diversos contextos, com maior propensão de consumo por parte das mulheres em situações de convívio e interação social, indicando um consumo como instrumento promotor da integração social e acadêmica^20,22^.

Os resultados obtidos ressaltam a importância de políticas públicas abrangentes e articuladas, de preferência preventivas, apostando na educação e promoção da saúde ou no incremento da qualidade de vida. Os dados evidenciam que as próprias instituições de ensino superior não podem ficar desresponsabilizadas diante da gravidade do fenômeno. Seu esforço em alertar para os efeitos fisiológicos e psicossociais de tal consumo e para a dependência criada, apelando à autonomia e responsabilidade dos jovens, parece esbarrar nas percepções formuladas pelos estudantes. Os serviços de consulta criados, incluindo ações de informação e de aconselhamento, não têm conseguido diminuir a incidência do consumo regular e dos picos em momentos festivos da academia. Numa lógica preventiva e relacionando o consumo abusivo a fragilidades na adaptação dos estudantes, é importante diminuir a frequência e a intensidade de situações de vulnerabilidade, de isolamento e de solidão que alguns jovens vivenciam no ensino superior, assim como assegurar contextos alternativos de interação social não marcados pelo consumo de substâncias. Nesse sentido, os dados oriundos a partir da investigação que utiliza um instrumento de *screening* válido sobre as percepções que os estudantes têm a respeito dos efeitos do álcool pode contribuir para implementação de estratégias de prevenção e promoção de saúde mais eficazes e condizentes com a realidade de cada universidade.
